# The Cytochrome P450 Superfamily Complement (CYPome) in the Annelid *Capitella teleta*

**DOI:** 10.1371/journal.pone.0107728

**Published:** 2014-11-12

**Authors:** Chris A. Dejong, Joanna Y. Wilson

**Affiliations:** Department of Biology, McMaster University, Hamilton, Ontario, Canada; Glasgow Caledonian University, United Kingdom

## Abstract

The Cytochrome P450 super family (CYP) is responsible for a wide range of functions in metazoans, having roles in both exogenous and endogenous substrate metabolism. Annelids are known to metabolize polycyclic aromatic hydrocarbons (PAHs) and produce estrogen. CYPs are postulated to be key enzymes in these processes in annelids. In this study, the CYP complement (CYPome) of the annelid *Capitella teleta* has been robustly identified and annotated with the genome assembly available. Phylogenetic analyses were performed to understand the evolutionary relationships between CYPs in *C. teleta* and other species. Predictions of which CYPs are potentially involved in both PAH metabolism and steroidogensis were made based on phylogeny. Annotation of 84 full length and 12 partial CYP sequences predicted a total of 96 functional CYPs in *C. teleta.* A further 13 CYP fragments were found but these may be pseudogenes. The *C. teleta* CYPome contained 24 novel CYP families and seven novel CYP subfamilies within existing families. A phylogenetic analysis identified that the *C. teleta* sequences were found in 9 of the 11 metazoan CYP clans. Two CYPs, CYP3071A1 and CYP3072A1, did not cluster with any metazoan CYP clans. We found xenobiotic response elements (XREs) upstream of *C. teleta* CYPs related to vertebrate CYP1 (CYP3060A1, CYP3061A1) and from families with reported transcriptional upregulation in response to PAH exposure (CYP4, CYP331). *C. teleta* had a CYP51A1 with ∼65% identity to vertebrate CYP51A1 sequences and has been predicted to have lanosterol 14 α-demethylase activity. CYP376A1, CYP3068A1, CYP3069A1, and CYP3070A1 were the most appropriate candidates for steroidogenesis genes based on their phylogeny and warrant further analyses, though no specific aromatase (estrogen synthesis) candidates were found. Presence of XREs upstream of *C. teleta* CYPs may indicate a functional aryl hydrocarbon receptor in *C. teleta* and candidate CYPs for studies of PAH metabolism.

## Introduction

The Cytochrome P450 (CYP) superfamily of protein enzymes are found in all domains of life [1], [2]. CYPs catalyze a monooxygenase reaction [3] of compounds that fall into two general categories: exogenous (i.e. xenobiotics) and endogenous (e.g. steroids and lipids) substrates. CYPs are involved in both the synthesis and catabolism of important biological signaling molecules. CYPs involved in metabolism of endogenous substrates typically act on a small number of very similar, structurally related molecules. CYPs responsible for metabolism of xenobiotics generally have more flexible active sites to allow them to act on a wider array of substrates.

All newly identified CYPs are named by the Cytochrome P450 nomenclature committee, using standard conventions for this gene superfamily. CYPs are named by amino acid sequence identity; genes with 40% and 55% identify are placed in the same family and subfamily, respectively [4]. CYPs are named by family and subfamily using a numeral and letter, respectively. The specific gene is given a number, by order of discovery [4]. For example CYP19A1 is in family 19, subfamily A and has a gene number of 1.

Since the early 2000's there have been several studies focused on the CYP genome complements (CYPomes) in metazoans, with studies completed on vertebrates [5]–[7], hemichordates [8], insects [9], crustaceans [10], and Cnidaria [11]. Many more CYPomes have been partially completed and unpublished CYPomes have been made available on the Cytochrome P450 webpage [12]. The smallest number of genes in a metazoan CYPome was found in the sponge *Amphimedon queenslandica* (35 CYP genes) and the largest metazoan CYPome identified so far included ∼235 genes in the lancelet, *Branchiostoma floridae* [13]. Vertebrate genomes typically contain 57–102 CYP genes [14].

Vertebrate steroidogenesis is well understood; the specific genes and proteins and the substrates and intermediates involved have been identified. CYPs and the hydroxysteroid dehydrogenases (HSDs) are the primary enzymes responsible for vertebrate steroidogenesis. The first step in the steroid pathway is the long-chain cleavage of cholesterol to pregnenolone via CYP11A [15]. The production of estradiol (18 carbon) from lanosterol (30 carbon) is a six to eight enzymatic step process and involves CYPs from families 11, 17, 19, 21 [15]. The sex steroids are one of the end products of steroidogensis. CYP19A has the aromatase function, which is responsible for estrogen production from androgen precursors. The CYP19A gene has only been found in chordates, though is predicted to have more ancestral origins [16].

*Capitella teleta* is a polychaete annelid found in marine environments along the Pacific and Atlantic shores around the continental United States, Japan and the Mediterranean [17]. There has been an interest in determining the identify and function of CYPs in *C. teleta*, primarily focused on deciphering their ability to metabolize xenobiotics and polycyclic aromatic hydrocarbons (PAHs; see [18], [19]). This stems from research that found *C. teleta* to be the most opportunistic invertebrate after a 1969 oil spill in Massachusetts [20] and a concentration dependent increase in CYP-dependent activity with exposure to PAHs [21] in *Capitella* spp, More recently, differences in tolerance to PAHs and capacity for PAH metabolism amongst *Capitella* species have been investigated [22], [23]. Two CYPs in *C. teleta*, CYP331A1 (a novel family) and CYP4AT1, have been identified and their expression was increased in response to various PAHs, suggesting a possible role of these CYPs in PAH metabolism [18].

Invertebrate endocrine systems have been much less studied that their vertebrate counterparts. Yet, data show that multiple endocrine active agents, sometimes including steroids typical in vertebrates, are present in invertebrate lineages [24]. Annelids are one group of invertebrates thought to produce and utilize the vertebrate sex steroid estradiol. *C. teleta* and *Platynereis dumerilii*, another marine annelid, had estrogen receptors (ERs) that responded to exogenous estrogen and regulated downstream gene expression, the first species with this function identified outside the vertebrates [25]. The annelid *Nereis virens* had detectable aromatase activity, likely occurring in the gut epithelium [26]. Despite having detectable aromatase activity, the protein responsible for this function remains unknown. CYPome studies in annelid species may provide clues to the evolution of the steroidogenesis pathway in metazoa and whether annelid invertebrates utilize the same enzymes for *de novo* sex steroid production.

The objective of this study was to annotate the *C. teleta* CYPome. The *C. teleta* CYPome is the first detailed analysis of a lophotrochozoan CYPome, providing important information on CYP content and evolution in an understudied metazoan superphyla. This study examines the potential role of the various CYPs in exogenous metabolism, particularly PAHs, and hypothesizes which CYPs may have a role in *C. teleta* steroidogenesis.

## Results

Eighty-four full length CYPs were identified and annotated from the *C. teleta* assembly (v1); the entire list of CYPs, their genomic location, size, and nomenclature are provided in Table S1. There were twelve partial CYP sequences identified that aligned well with existing ESTs but could not be completed based on the current assembly (Table S2). There were thirteen partial CYP sequences identified that lacked any EST support (Table S3); whether these were genes or pseudogenes remains unclear. Based on the names assigned by the cytochrome P450 nomenclature committee, the predicted *C. teleta* CYPs were found in 9 of the 11 known metazoan CYP clans [13] and predicted 24 novel CYP families and 7 novel CYP subfamilies.

All of the full length CYPs contained at least some signature CYP motifs (Tables 1 and 2; Tables S4 and S5). The I-helix motif [A/G]GX[D/E]T[T/S] [27]; had conservation of at least three of the six amino acids in all but thirteen *C. telata* CYPs (CYP3065A1–4, CYP3065B1, CYP3066A1–3, CYP3066C1, CYP3067A1, CYP372B1, and CYP39B1). The remaining CYPs had obvious sequence homology, with a majority of the conservation at the ends in the I-helix motif, even though this is the most poorly conserved motif of the four examined. The K-helix motif was fully conserved across all of the *C. teleta* sequences with no exceptions to the E-X-X-R consensus sequence (Table 1) [27]. The meander coil was conserved across all of the annotated sequences, although CYP372B1 and CYP4EE1 has substitutions for the first two amino acids in the motif (Table 1). Lastly, the cysteine residue in the heme binding loop is highly conserved, with very few exceptions [28], and this residue was present in all of the *C. teleta* sequences (Table 2). There was clear homology in the heme loop motif across all of the *C. teleta* sequences except for a gap in the motif in CYP3067A1. Interestingly, CYP3067A1 had a gap in both the heme loop and I-helix motifs (Table 2).

**Table 1:** Subset of highly conserved motifs across the *Capitella teleta* CYPome.

	K-helix		Meander Coil	
CYP	AA	EXXR	AA	FDPER
**CYP10B1**	341	**E**TF**R**	393	**F**K**PER**
**CYP20A1**	338	**E**SL**R**	390	**FDPER**
**CYP26D1**	348	**E**VL**R**	400	**FDP**D**R**
**CYP3052A1**	362	**E**LL**R**	415	**F**E**PER**
**CYP3052A10**	365	**E**LL**R**	418	**F**Q**PER**
**CYP3052A2**	361	**E**LL**R**	414	**F**E**PER**
**CYP3052A3**	361	**E**LL**R**	414	**F**E**PER**
**CYP3052A4**	349	**E**LL**R**	378	..**PER**
**CYP3062A1**	359	**E**VY**R**	412	**F**N**P**DN
**CYP3062A2**	372	**E**VY**R**	425	**F**N**P**N**R**
**CYP3063A1**	358	**E**IM**R**	411	**F**N**P**D**R**
**CYP3064A1**	335	**E**VL**R**	388	**F**N**P**S**R**
**CYP3065A1**	379	**E**TY**R**	432	**F**R**PER**
**CYP3065A2**	336	**E**TY**R**	389	**F**R**PER**
**CYP3065A3**	336	**E**TY**R**	389	**F**R**PER**
**CYP3065A4**	377	**E**CY**R**	430	**F**K**PER**
**CYP3065B1**	375	**E**TF**R**	428	**F**K**PER**
**CYP3066C1**	380	**E**SL**R**	432	**F**N**P**K**R**
**CYP3067A1**	344	**E**SF**R**	400	**F**KYD**R**
**CYP3068A1**	336	**E**ML**R**	388	**FDP**Y**R**
**CYP3069A1**	312	**E**TL**R**	364	**F**N**P**DQ
**CYP3070A1**	366	**E**TL**R**	418	**F**N**P**D**R**
**CYP331A2**	363	**E**TL**R**	418	**F**E**PER**
**CYP371B1**	403	**E**AL**R**	455	**F**I**PER**
**CYP372A1**	346	**E**SF**R**	391	**F**I**PER**
**CYP39B1**	347	**E**SI**R**	398	**F**K**P**D**R**
**CYP44C1**	359	**E**GF**R**	411	**F**I**PER**
**CYP4AT1**	356	**E**SL**R**	409	Y**DPER**
**CYP4BK4**	304	**E**SM**R**	356	**F**R**P**D**R**
**CYP4EE1**	399	**E**SL**R**	452	YN**PER**
**CYP4V25**	367	**E**TL**R**	419	**F**I**P**D**R**
**CYP51A1**	364	**E**TL**R**	416	**F**N**P**D**R**

Two motifs (K-helix, and meander coil) are represented in an aligned format to show conservation across the *C. teleta* CYPs. Bolded letters represent conserved residues. AA is the amino acid number where the motif begins in each gene. The expected motif sequence is given in each heading for comparison. The glutamic acid and arginine residues in the meander coil are conserved across the entire CYPome.

**Table 2:** Subset of less conserved motifs across the *Capitella teleta* CYPome.

	I-helix		Heme Loop	
CYP	AA	[A/G]GX[D/E]T[T/S]	AA	PFXXGXRXCXG
**CYP10B1**	284	**G**AV**ETT**	415	**PF**GH**G**A**R**M**C**I**G**
**CYP20A1**	281	**AG**FH**TT**	410	**PF**GF**G**K**R**K**C**L**G**
**CYP26D1**	289	**AGYETT**	421	**PF**GS**G**S**R**S**C**A**G**
**CYP3052A1**	307	**AG**TA**TT**	440	**PF**GA**G**P**R**V**C**L**G**
**CYP3052A2**	307	**AG**TA**TT**	439	**PF**GA**G**P**R**V**C**M**G**
**CYP3052A3**	308	**GG**TA**TT**	439	**PF**GA**G**P**R**V**C**L**G**
**CYP3052A4**	295	**AG**TS**TT**	401	**PF**GA**G**P**R**V**C**L**G**
**CYP3062A2**	319	**AG**T**E**SM	447	**PF**GA**G**M**R**R**C**P**G**
**CYP3063A1**	301	**AG**T**E**TS	437	**PF**GA**G**K**R**K**C**I**G**
**CYP3064A1**	281	**G**VS**D**G**S**	410	**PF**ST**G**Q**R**S**C**V**G**
**CYP3065A1**	322	DSLD**T**L	452	**PF**GV**G**P**R**S**C**P**G**
**CYP3065A2**	279	DSL**DT**L	409	**PF**GV**G**P**R**S**C**P**G**
**CYP3065A3**	279	DSLD**T**L	409	**PF**GV**G**P**R**S**C**V**G**
**CYP3065A4**	320	DAL**D**SL	450	**PF**GL**G**P**R**A**C**A**G**
**CYP3066C1**	323	S**G**HS**T**V	452	**PF**GM**G**P**R**S**C**I**G**
**CYP3067A1**	286	....N**T**	424	A**F**GS…L**C**P**G**
**CYP3068A1**	278	**A**SQ**ET**L	411	**PF**GA**G**N**R**T**C**V**G**
**CYP3069A1**	264	**G**AQE**T**L	383	**PF**GG**G**AHA**C**V**G**
**CYP3070A1**	309	**AG**Q**ETT**	436	**PF**SL**G**Q**R**S**C**L**G**
**CYP331A2**	306	**AG**Y**DTT**	438	**PF**GA**G**P**R**N**C**I**G**
**CYP371B1**	346	**G**AV**DTT**	476	**PF**GF**G**A**R**S**C**I**G**
**CYP372A1**	281	**AG**I**D**S**T**	414	**PF**GY**G**P**R**M**C**I**G**
**CYP372B1**	280	PNI**E**IEDRS**T**	421	**PF**SH**G**L**R**A**C**P**G**
**CYP39B1**	282	**A**SLANA	419	**PF**GG**G**RFQ**C**P**G**
**CYP44C1**	301	D**G**MI**TT**	432	**PF**SC**G**P**R**M**C**P**G**
**CYP4AT1**	299	E**G**H**DTT**	429	**PF**SA**G**P**R**N**C**I**G**
**CYP4EE1**	340	E**G**H**DTT**	472	**PF**SA**G**P**R**N**C**I**G**
**CYP4V25**	308	E**G**H**DTT**	439	**PF**SA**G**L**R**N**C**I**G**
**CYP51A1**	306	**AG**QH**TS**	437	**PF**GA**G**RHR**C**I**G**

Two motifs (I-helix, and heme loop) are represented in an aligned format to show conservation across the *C. teleta* CYPs. Bolded letters represent conserved residues. AA is the amino acid number where the motif begins in each gene. The expected motif sequence is given in each heading for comparison. The cysteine residue in the heme loop are conserved across the entire CYPome. Note the lack of conservation in CYP372B1 (I-helix) and CYP3067A1 (I-helix and heme loop).

**Figure 1:**
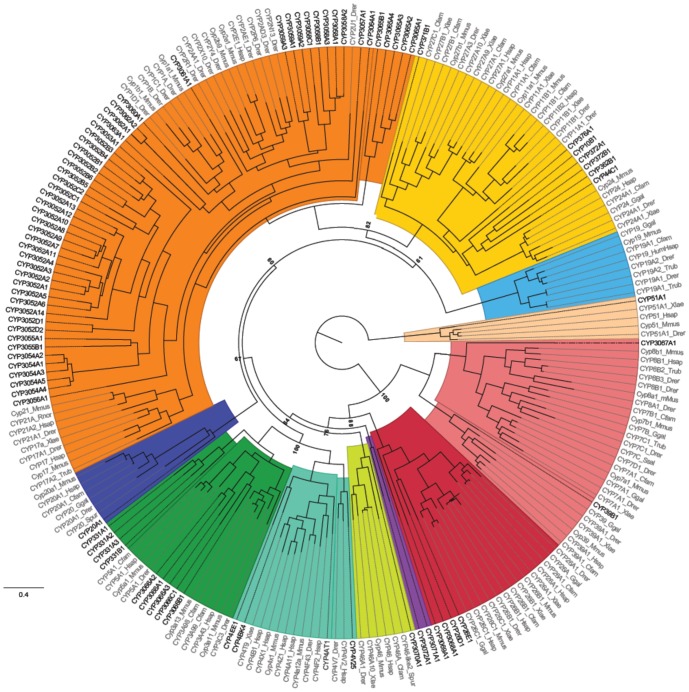
Phylogenetic tree of Cytochrome P450s in metazoa. The tree was completed on RaxML using non-parametric bootstrapping with a gamma distribution. The tree was rooted with CYP51. The black names are the *Capitella teleta* sequences. The tree is colour coded by clan: clan 2 orange, clan 3 dark green, clan 4 teal, clan 7 salmon, clan 19 light blue, clan 20 dark blue, clan 26 red, clan 46 lime green, clan 51 beige, mitochondrial clan yellow, and the two sequences that do not fit into a clan (CYP3071A1 and CYP3072A1) are purple.

**Figure 2:**
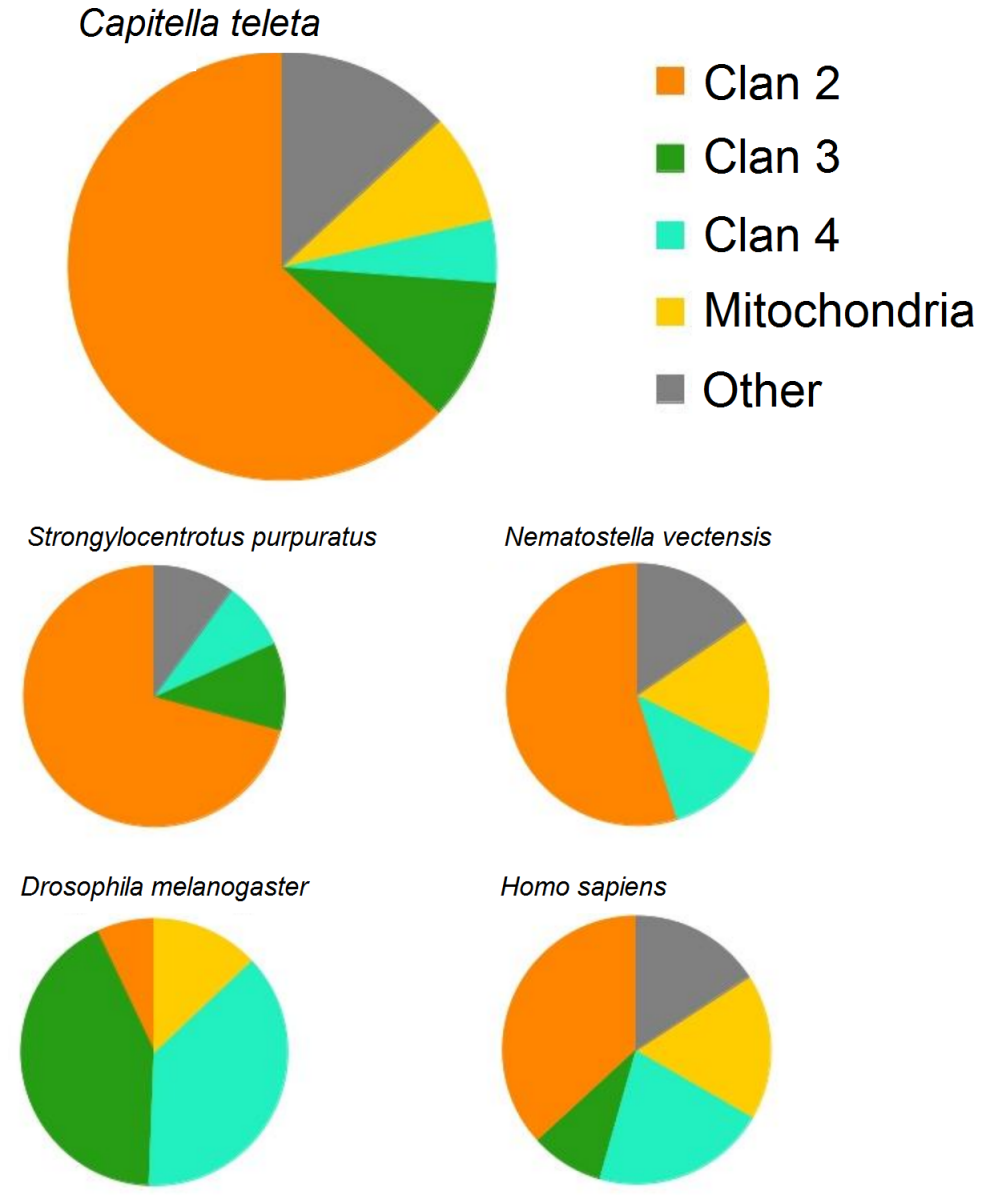
Distribution of the major Cytochrome P450 clans in five different species. *Capitella teleta, Strongylocentrotus purpuratus* [8], *Nematostella vectensis* [11], *Drosophila melanogaster* [9], and *Homo sapiens* [5] are compared.

The phylogenetic relationships among the genes of the *C. teleta* CYPome is shown in Figure 1 and the distribution of these genes in the major clans (clans 2, 3, 4, and mitochondrial) shown in Figure 2. A majority of the *C. telata* CYPs were in clan 2, accounting for ∼60% of the CYPome (Figure 2); Six of these genes were the most basal sequences within this clan (Figure 1). 33 genes were clustered as a distinct sister group to the CYP1 and CYP2 genes, without a single vertebrate sequence (Figure 2). Five sequences clustered with the vertebrate CYP1s and eight sequences were clearly clustered within the CYP2s. As expected, CYP2U1 was the most basal of the CYP2 genes (Figure 2). There were no *C. telata* sequences that clustered with the CYP17 or CYP21 sequences.

Clan 3 and 4 contained nine and four CYPs, respectively, while six genes were from the mitochondrial clan (Figure 2). A single *C. telata* sequence was found to cluster with CYP4V (CYP4V25), CYP7s/CYP306s (CYP3067A1), CYP11A (CYP376A1), CYP20 (CYP20A1), CYP27s (CYP371B1), CYP39 (CYP39B1), CYP46 (CYP3070A1), CYP51 (CYP51A1). Interestingly, there were a small numbers of genes (4 sequences) that clustered with CYP26s (Figure 1).

**Figure 3:**
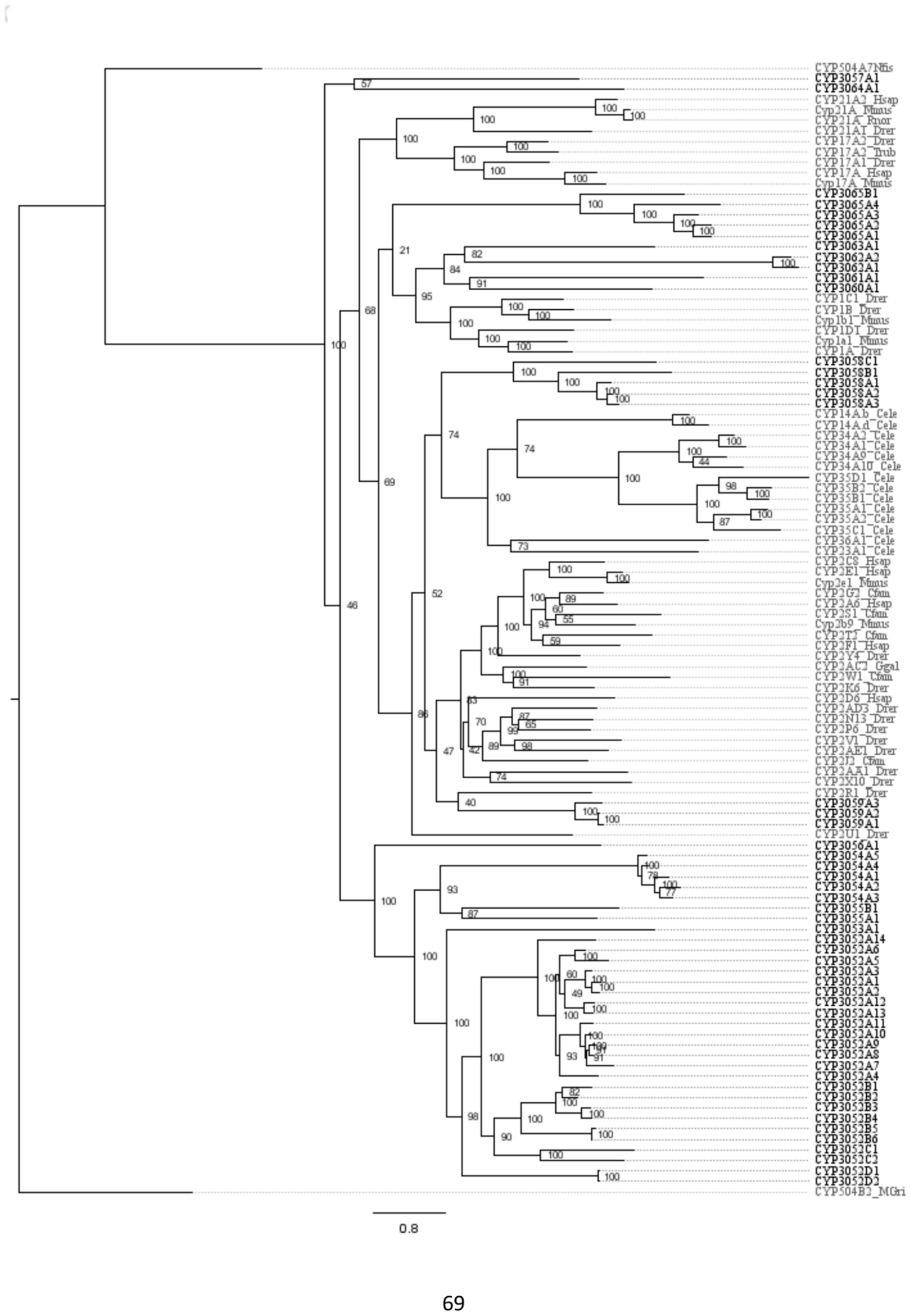
Phylogeny of Cytochrome P450 clan 2. The sequences are identical to those in Figure 1 with added invertebrate sequences to increase internal node resolution. The tree was completed on RaxML using non-parametric bootstrapping with a gamma distribution. *C. teleta* sequences are in black, all other sequences are in gray. The phylogeny was rooted using fungal CYP86s.

**Figure 4:**
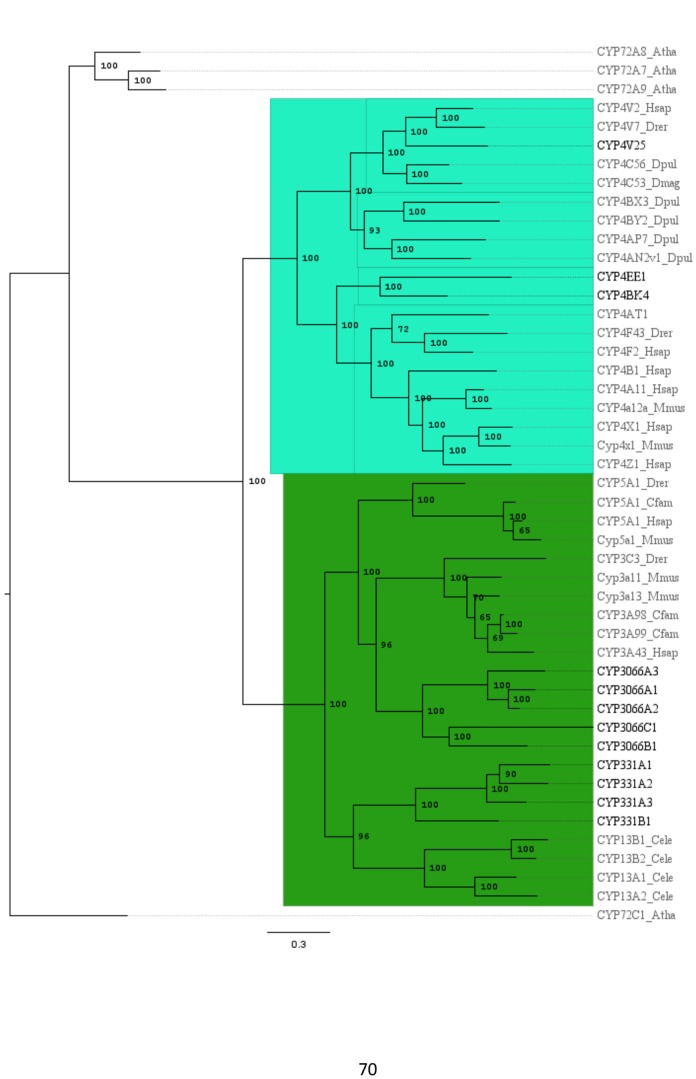
Phylogeny of Cytochrome P450 clan 3 and 4. The sequences are identical to those in Figure 1 with added invertebrate sequences to increase internal node resolution. The tree was completed on RaxML using non-parametric bootstrapping with a gamma distribution. *C. teleta* sequences are in black, all other sequences are in gray. The phylogeny was rooted using fungal *Arabidopsis thaliana* CYP72s.

**Figure 5:**
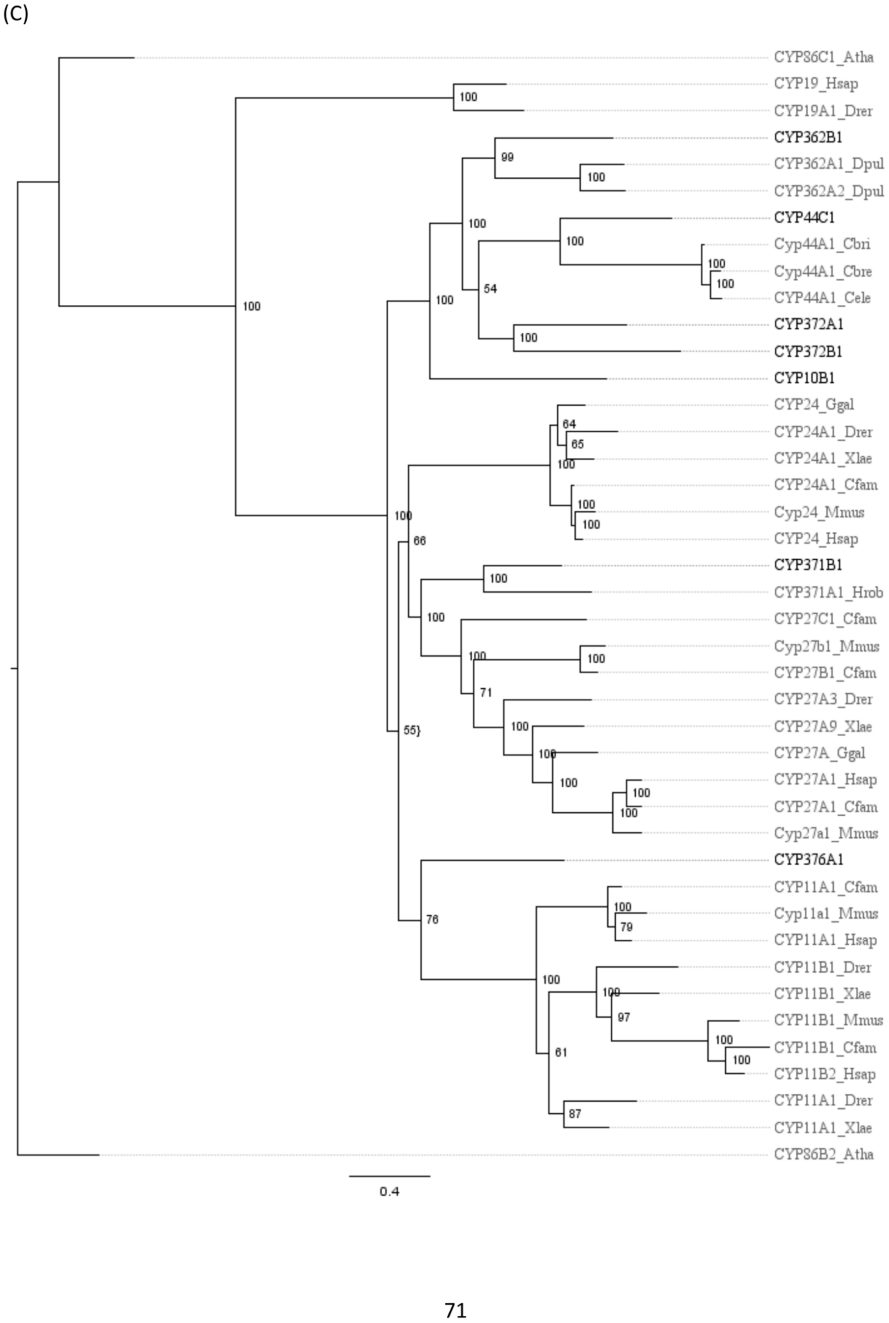
Phylogeny of Cytochrome P450 mitochondrial clan. The sequences are identical to those in Figure 1 with added invertebrate sequences to increase internal node resolution. The tree was completed on RaxML using non-parametric bootstrapping with a gamma distribution. *C. teleta* sequences are in black, all other sequences are in gray. The phylogeny was rooted using CYP19s and *Arabidopsis thaliana* CYP86s.

Figures 3, 4, and 5 shows phylogenies for clans 2 (Figure 3), 3 and 4 (Figure 4), and the mitochondrial clan (Figure 5). Invertebrate sequences were added to those sequences included in Figure 1 to help resolve and increase bootstrap support for internal branching arrangements within each clan (Figures 3–5). The addition of invertebrate sequences to the larger phylogeny interfered negatively with tree construction, producing a phylogeny with less robust bootstrap values. In the clan 2 phylogeny (Figure 3), sequences from *C. elegans* and *D. pulex* were added to the analysis; the *C. elegans* sequences clustered with the CYP2s. The CYP3058 family, clustered closest with the *C. elegans* sequences. The large cluster of clan 2 *C. teleta* CYPs remained on their own, as in the large phylogeny (Figure 1), basal to the rest of the clan 2 sequences. Clan 3 and 4 were sister clans (Figure 1) and were included together on the same clan phylogeny (Figure 4) with added sequences from *C. elegans*, *H. robusta* and *D. pulex*. The additional *C. elegans* sequences clustered closest with the CYP331 family, which were basal in clan 3 in the large phylogeny (Figure 1). The *D. pulex* sequences clearly clustered with the CYP4Vs, including the *C. telata* CYP4V25 (Figure 4). In the mitochondrial clan phylogeny (Figure 5), CYP10B1, CYP362B1, CYP44C1, CYP372A1, and CYP372B1, clustered with CYP36 from *D. pulex* and CYP44 from *C. elegans*. CYP371B1 clustered with *H. robusta* CYP371A1.

Table 3 provides the upstream XREs of *C. teleta* genes from CYP families CYP331 and CYP4. The CYP1-like genes, CYP3060A1 and CYP3061A1, were also examined. These CYP genes were either closely related to vertebrate CYP1s (CYP3060A1, and 3061A1) or genes that were upregulated in response to PAH exposure (CYP331 and CYP4 [18]. CYP331A1 had three XREs within 10 kb of the start site, the remaining CYP331A genes had no XREs. CYP331B1, CYP3060A1, CYP3061A1, and CYP4AT1 each had one XRE 10 kb upstream. Multiple XREs were found upstream of CYP4V25 (two) and CYP4BK4 (four). Only CYP4EE1, of the *C. teleta* CYP4s, had zero XREs upstream of the start site. There was a p-value of 5.96e−05 and q-value of 1 for each of the sites, this was calculated using overall genome base frequencies.

**Table 3:** Xenobiotic response elements upstream of *C. teleta* CYPs.

CYP	Number of XREs 10 kb upstream
CYP331A1*	3
**CYP331A2**	0
**CYP331A3**	0
**CYP331B1**	1
**CYP4V25**	2
CYP4AT1*	1
**CYP4BK4**	4
**CYP4EE1**	0
**CYP3060A1**	1
**CYP3061A1**	1

Each gene was searched for the consensus xenobiotic response element (XRE) sequence TNGCGTG [39], 10 kb upstream of the start site. Genes were chosen based on homology to vertebrate CYP1s and genes from clan 3 and 4. Genes were searched in the 10 kb upstream region. The asterisks mark the genes which were transcriptionally upregulated with exposure to the PAHs benzo[α]pyrene, 3-methylcholanthrene, or fluoranthene [18]. p-value of 5.96e−05 and q-value of 1 for all sites.

## Discussion

### CYPome Annotation

Annotation of CYPomes can be challenging when working in species that are distantly related to those with a defined CYPome, because the searches are based on homology to known, yet distant sequences: *C. teleta* is the first lophotrochozoan to have its CYPome annotated and vertebrate sequences were primarily used in our initial searches. These reference vertebrate sequences were well curated, with very high confidence in their annotation, including exon boundaries, making any manual corrections from the PASA output for *C. teleta* more reliable. Annotations of *C. teleta* were additionally verified using *C. elegans* and *D. pulex* sequences for unique hits and no significant regions were found that the vertebrate sequences missed. Overall, our analysis predicted eighty-four full length CYPs, and identified twelve partial CYP sequences that aligned well with existing ESTs, and thirteen partial CYP sequences that lacked any EST support. Our analysis of the *C. teleta* CYPome has identified 24 novel families and 7 novel subfamilies. CYP26 contained two new subfamilies and CYP4, CYP10, CYP39, CYP44, CYP352 each had one new subfamily in *C. teleta*. The CYPomes of non-chordate phyla often contain novel CYP families [12], [14]: *C. elegans* contained 14 unique families [12] and the *D. melanogaster* CYPome contained 24 families with most families unique to arthropods [9].

During manual annotation it was important to ensure that genes had a length of ∼1500 bp, the average length for a CYP. Start (ATG) and stop (TAA/TAG/TGA) codons were noted and always present, as well as appropriate splice signals (GT/AG) [29] at intron/exon boundaries. The numbers of exons were not well conserved between related CYPs in other species. Exon number was taken into consideration between related sequences within *C. teleta* during searches for missing exons where EST data was lacking for annotation.

All of the fully annotated *C. teleta* CYPs had EST support covering all or almost all of the gene. A notable exception was CYP3052C1, which was missing EST data for exons one and two. Homology searches in the expected upstream and downstream regions were able to identify the missing exons. There were 12 incompletely annotated CYPs with EST support and these were presumed to be functional, full length genes though they could not be resolved with the existing genome assembly. Thus, the total number of CYPs identified in *C. telata* was 96, which fall into the range predicted by Nelson and colleagues [13] and is comparable to the 50–100 genes found in vertebrate CYPomes [5]–[7], 120 genes in the sea urchin *S. purpuratus* [8], 75 genes in the crustacean *Daphnia pulex* [10], 83 genes in the insect *D. melanogaster* [9] and 82 genes in the sea anemone *N. vectensis* [11]. *C. teleta* had an average number of CYPs for a metazoan CYPome.

There were 13 gene fragments that may be pseudogenes. These fragments lacked EST support or had identifiable early stop codons. The number of possible pseudogenes per functional gene (.14) is higher than noted in other species: *Daphnia pulex* has.04 [10], *C. elegans* has.1 [12] and *D. melanogaster* has.08 [9] pseudogene per gene. It is possible that a small number of these fragments were functional genes. One CYP on scaffold 342 had EST support but had an in frame stop codon in the first exon.

To provide support for the annotation process, the identified CYPs were examined for conserved CYP motifs. The heme binding region starts around amino acid 430 and has a well conserved motif of PFXXGXXRXCXG (the 'X' represents a non conserved amino acid); the cysteine, until recently, was considered the only absolutely conserved amino acid in all known CYPs, although exceptions have been documented [28]. There are three other well conserved motifs: portions of the I-helix, [A/G]GX[E/D]T[T/S], located around amino acid 300; K-helix, EXXR, located around amino acid 360; and an area known as the ‘meander coil’, FDPER, located around amino acid 410 [30]. The K-helix motif is incredibly well conserved in CYPs, with only a handful of known exceptions to the two conserved amino acids [31]. These motifs are important when analyzing potential CYPs, if one or more of these regions are missing, or out of place, it is likely that the gene was constructed incorrectly, a pseudogene or not a CYP at all.

The high conservation in the motifs (Tables 1 and 2) was expected and supports our annotation of these genes as CYPs. The least conserved domain is the I-helix, and our findings in *C. teleta* support this; CYP3065A1–4, CYP3065B1, CYP3066A1–3, CYP3066C1, CYP3067A1, and CYP372B1, CYP39B1 all have lower conservation in the I-helix. The gaps in CYP3067A1 and the insertion in CYP372B1 are peculiar. Whether these genes are fully functional may be questioned, yet, there is EST support to show that they are expressed.

The *C. teleta* CYPome phylogenetic analysis (Figure 1) contains almost exclusively vertebrate sequences, along with the *C. telata* sequences we identified. The arrangement of the clans was consistent with previous work, down to the family level of the known sequences [6], [7]. It was difficult to add any sequences outside of vertebrates because of their divergence from vertebrate and *C. teleta* sequences and the lack of sequences that would help provide definitive phylogenetic relationships. When *Drosophila melanogaster* sequences were added to the phylogeny the bootstrap support was very weak, especially in clan 2–4 where most *D. melanogaster* sequences were added, and this is likely due to the evolutionary distance between vertebrates, insects and annelids. The *D. melanogaster* CYPome paper [9] provides a prime example of the difficulty in creating phylogenies between vertebrates and invertebrate CYP sequences. There were major branches (i.e. those that separate clans) with less than 10% bootstrap support [9]. The more recent *D. pulex* CYPome [10] had much better support at the clan level (support beyond the clan level was not provided), which was due to increased saturation in arthropod CYPs from available insect CYP sequences and basal chordate CYPs. As genome sequences become available from a wider array of species across the major metazoan phyla, the evolutionary distance between CYPomes will be reduced and help improve the phylogenetic analyses.

The clan phylogenies (Figures 3–5) have additional invertebrate sequences to help resolve nodes within the major clans found in the *C. teleta* CYPome. The phylogenies are rooted using plant and fungi CYPs, these sequences were the closest CYPs to the clans that were being rooted on the CYP webpage [12]. Using closely related clans as an outgroup, such as clan 46 for the clan 3 and 4 phylogeny, did not provide robust bootstrap support. The clan 2 phylogeny (Figure 3) included sequences from *C. elegans*, the clan 3 and 4 phylogeny (Figure 4) included *D. pulex, D. magna,* and *C. elegans*. sequences, and the mitochondrial phylogeny (Figure 5) incorporated sequences from *D. pulex, Caenorhabditis spp.*, and *H. robusta*. The addition of these sequences in the clan phylogenies increased the support for the internal nodes (data not shown). The sequence similarity data and phylogenetic analyses have provided information to infer the placement of the *C. teleta* CYPs into clans and assign nomenclature.

### Clan Distribution

*C. teleta* possesses CYPs from all the metazoan clans except for 19 and 74. Clan 19 has not been found outside chordates [32] and clan 74 had not been found outside anemone and placozoa [13], although it has been recently found in amphioxus [16]. *C. teleta* is the first protostome analyzed to have representation in clan 46. CYP46 is the only clan 46 CYP gene in vertebrates and functions as a cholesterol 24-hydroxylase in the brain [33]. Since *C. teleta* CYP3070A1 had only 35% identity with human CYP46A1, it is difficult to predict whether the function is conserved in the *C. telata* ortholog. *In silico* molecular docking or 3D modeling of the protein may help support or refute the possibility that cholesterol is a substrate of CYP3070A1.

There are 53 full length clan 2 CYPs in *C. teleta*, representing ∼60% of the total CYPome (Figure 2). This is the second largest in relative size for known CYPomes and is smaller only to *S. purpuratus* (∼70%, Figure 2). Insects generally have only 5.5–10% of their CYPs in clan 2 [10]. The function of many insect clan 2 CYPs are unknown but some are known for their role in ecdysone synthesis [10]. Clan 2 CYPs are much more important for metabolism of exogenous compounds in mammals [34].

All of the *C. telata* clan 2 CYPs were located in novel CYP families; indeed the *C. telata* clan 2 sequences had 14 novel CYP families made up from 20 subfamilies. It has been postulated that a large number of CYPs related to families involved in exogenous metabolism (i.e. families 1–4) may suggest evolutionary pressure towards diverse function [11]. The largest family was CYP3052 with 24 sequences; these sequences made up the majority of the large standalone cluster of 33 *C. teleta* CYPs on the phylogenetic tree (Figure 1). If this family of *C. teleta* CYPs follows the trend of other large CYP families, namely families CYP1–4, then these proteins may be involved in xenobiotic metabolism.

There were five novel families with a single sequence each (CYP3060–3063 and CYP3065) that were CYP1-like. There were fewer CYP1-like genes in *C. telata* than were found in *S. purpuratus* (11) but similar to what is typical (3–4 CYP1 genes) in vertebrates [8], [12]. Of the two families that grouped with CYP2s, CYP3058 clustered more closely with *C. elegans* sequences than vertebrate CYP2 sequences in the clan 2 phylogeny (Figure 3). The other family, CYP3059, clustered with vertebrate CYP2R, although the bootstrap support in the clan phylogeny was quite low (Figure 3) suggesting the placement of this family is uncertain with respect to the vertebrate CYP2 families. The function of the *C. elegans* CYPs are unknown but vertebrate CYP2s are well known for their role in xenobiotic metabolism [34]. CYP3057A1 and CYP3064A1 were basal in this clan and had high divergence from the remaining sequences.

The clan 3 phylogeny had sequences from across all metazoan phyla. Clan 3 contains families CYP3 and CYP5 in vertebrates, but is represented by different families in invertebrates such as families CYP6 and CYP9 [35]. Mammalian CYP3s are known to have very flexible active sites that can accommodate structurally diverse substrates. CYP3A4 is the most important enzyme involved in drug metabolism in humans but other CYP3s are also important in metabolism of endogenous and exogenous compounds [33]. Clan 3 CYPs are involved in both endogenous and exogenous metabolism in arthropods [10]. *C. teleta* had two clan 3 families with a total of nine CYPs; both families were novel. *N. vectensis* had 20 clan 3 CYPs [11], *S. purpuratus* had 10 [8], and *D. melanogaster* has an expanded clan 3 with 36 CYPs [9]. Mammals appear to have a much smaller number of clan 3 genes than many invertebrate species; humans have just five clan 3 sequences from a single subfamily [5].

The *C. teleta* clan 3 sequences included CYP331A1, which had been previously described [18]. CYP331A1 had increased expression from exposure to benzo[α]pyrene (BaP) and fluoranthene, two PAHs [18]. The CYP331 family has been expanded in this annotation with two more *CYP331A* genes and the *CYP331B1* gene.

The CYP4 family was expanded in *D. melanogaster* (32) and other insects [9], but was relatively limited in *N. vectensis* (3) [11]. There were five clan 4 CYPs in *C. teleta* and all were from the CYP4 family. CYP4V25 was an ortholog to CYP4Vs yet was below the 55% sequence identity threshold used during standard nomenclature. All of the top BLAST hits for CYP4V25 were CYP4Vs from various species (data not shown). Furthermore, CYP4Vs have been found in molluscs and crustaceans (DR Nelson, personal communication). Collectively, this information supports the placement of this sequence into the CYP4V family despite the low sequence identity to other gene members. Little is known of CYP4 function outside vertebrates. CYP4C has a role in juvenile hormone synthesis in the cockroach *Blaberus discoidalis* [36]. In vertebrates, CYP4s primarily metabolize endogenous compounds, specifically fatty acids, although they do metabolize some exogenous pharmaceuticals (e.g. erythromycin) [37]. Yet, even in mammals the function of CYP4V is unknown [33].

Like the CYP4Vs, the function of CYP20A1 remains unclear in vertebrates. The *C. teleta* CYP20A1 is ∼40% identical to other CYP20A1s but is a clear ortholog (Figure 1) with no other closely related sequences. CYP20A1 has been documented in invertebrates such as *S. purpuratus* and *H. robusta* [13]. It is interesting that CYP20A1 has unknown function yet has such clear homology between annelids and vertebrates.

CYP10 has been identified in molluscs and has been suggested as the only family in the mitochondrial clan in molluscs [38]. Since orthologs have now been identified in two major phyla, the *C. telata* CYP10 may suggest that CYP10 is present in all lophotrochozoans. Interestingly, CYP10 was not the only mitochondrial CYP in *C. telata*. CYP44 was placed in the mitochondrial clan; a CYP44 homolog has also been found in *C. elegans* [12], roundworms and molluscs [13]. Thus, CYP10 and CYP44 may be expected mitochondrial CYPs in lophotrochozoans.

### PAH and xenobiotic metabolism

*C. teleta* has been long known to metabolize PAHs in sediment, and it has been suggested that CYPs were responsible [39], [40]. BaP was metabolized, likely by CYPs, in another annelid, *Nereis virens* [40]. There is conflicting data on whether CYPs are transcriptionally upregulated after PAH exposure in *N. virens* (primarily tested with BaP exposure), with some studies reporting a 2-fold increase in CYPs and others reporting no change (reviewed by [40]). In *C. teleta*, two CYPs (CYP4AT1 and CYP331A1) had a 1.9–2.6 fold increased expression with exposure to some PAHs, including BaP [18]. CYP4AT1 had 1.25 to 1.9 fold increase in gene expression after exposure to PAH contaminated sediments [18]. Interestingly, there were 3 genes found for *C. teleta* in the CYP331 family, two of which were in the same (CYP331A) subfamiliy.

An important factor to consider is the presence of CYP co-enzymes in these reactions such as cytochrome P450 reductase and cytochrome b5 [41]. The current putative JGI *C. teleta* transcriptome assembly predicts the presence of cytochrome P450 reducatase (estExt_Genewise1Plus.C_990037) and cytochrome b5 (estExt_Genewise1.C_2000018), which shows the presence of co-enzymes necessary for functional CYP activity in this species.

Vertebrates, including mammals, mediate metabolism of PAHs through CYP1A and CYP1 gene expression is increased with exposure to PAHs (reviewed in [43]), through transcriptional activation via the aryl hydrocarbon receptor (AHR) pathway [44]. In mammals, the CYP2B and CYP2C subfamilies are also important for PAH metabolism [42] but these subfamilies are not present in all vertebrates. The AHR is activated by planar PAHs and halogenated aromatic hydrocarbons; TCDD is the ligand with the highest affinity for this receptor in many species [45]. AHRs transcriptionally regulate a battery of genes through interaction with a specific sequence, the xenobiotic or dioxin response element (XRE or DRE) [46]. Many AHR ligands, including PAHs, are also substrates for CYP1 enzymes [45]. AHRs are present in invertebrates and the amino acid sequence of the DNA binding domain is similar to that found in vertebrates. Indeed, AHRs from *Drosophila* [47], *C. elegans* [48], and *Mya arenaria* [49] are capable of binding with the mammalian XRE sequence. Therefore, we examined the upstream region of the CYP1-like (CYP3060A1 CYP3061A1), CYP331 and CYP4AT genes in *C. telata* to determine if XREs were present (Table 3). CYP331A1 had three XREs within 10 kb of the start site, but CYP4AT1 had only one. The difference in the number of XREs between these two CYPs may explain the difference in expression during BaP exposure, since there is a relationship between the number of XREs and the relative upregulation of the gene [50]. The CYP1-like *C. teleta* sequences had one XRE and CYP4BK4 had four XREs in the 10 kb upstream region. Should the AHR have a role in regulating gene transcription in *C. teleta* after exposure to PAHs, we would predict that CYP4BK4 would have the greatest transcriptional response. Considering the structural link between AHR ligands and CYP1 substrates in vertebrates, we might speculate that CYP4BK4 be a primary candidate gene for studies of PAH metabolism in this species. Future PAH exposure studies in *C. teleta* will shed light on to role of the AHR and XREs in *C. teleta* and the potential role these CYPs may play in PAH metabolism.

### Steroidogenic CYPs

CYP51A1 enzymes are responsible for lanosterol-14-alpha-demethylation; the conversion of lanosterol into cholesterol [51]. Cholesterol is the precursor to steroids and this function is expected in all species with endogenous steroid production. The next step in vertebrate steroidogenesis is cholesterol-side-chain-cleavage, which is completed by CYP11A1 in vertebrates and converts cholesterol to pregnenolone [15]. There was one *C. teleta* CYP (CYP376A1) that clustered with the CYP11 family in the phylogenetic tree (Figure 1) and is the best candidate for cholesterol side-chain-cleavage function in *C. teleta*. CYP11B functions in the synthesis of cortisol and coticosterone [15], [52], which are not expected in annelids since these molecules have not been found in amphioxus, *Ciona intestinalis*, or sea urchins [53].

CYP17A1 functions as a 17-alpha-hydroxylase, which is responsible for converting pregnenolone into DHEA. The production of DHEA is the next step in steroidogenesis after side-chain-cleavage and before the production of androgens [15]. Since there is no *C. telata* CYP that clusters with the CYP17 genes from vertebrates, it is difficult to predict which CYP is likely to complete this function at this time. There were many clan 2 CYPs identified but whether 17-alpha-hydroxylase activity is mediated by one of them is unclear. Analyzing the single copy clan 2 CYPs (e.g. CYP3057A1 and CYP3064A1) would be an appropriate place to begin the search for a 17-alpha-hydroxylase enzyme.

Detectable estrogen production has been documented in annelids [26], yet there was no CYP19 identified in the *C. teleta* CYPome. This is not surprising, as a CYP19 has not been identified outside of chordates and sea anemone had no CYP19 [11], in spite of endogenous estrogen production [54]. It has been postulated that another CYP has the aromatase function outside of chordates [11]. There are many CYPs identified in *C. teleta*, the most promising candidates genes for steroidogenic functions are the single copy CYPs from clan 2, CYP376A1 from the mitochondrial clan, CYP3068A1 or CYP3069A1 from clan 26 and CYP3070A1 from clan 46. All of these CYPs should be further examined by *in silico* methods for their potential ability to bind the intermediates of the steroidogenic pathway.

### Conclusion

*Capitella teleta* has an interesting complement of CYPs. CYPs were found in nine of the eleven metazoan CYP clans. There were a total of 24 novel CYP families; careful study will be required to determine their function. The annotation of the *C. teleta* CYPome will make annotating other lophotrochozoan CYPomes easier. With additional annelid and other lophotrochozoan CYP sequences, we will better understand which of the novel CYP families and subfamilies discovered here are specific to annelids. *C. teleta* is known to survive well in polluted environments and two CYP genes CYP331A1 and CYP4AT1 were known to be transcriptionally regulated by PAHs [18]. Indeed, several more closely related homologs were identified in this study. XRE sequences were found upstream in several of these genes suggesting that CYP331A1, CYP331B1, several CYP4s and the CYP1-like CYP3060A1 and CYP3061A1 genes may be in the AHR gene battery. Empirical testing will be needed to demonstrate this and explore their possible role in PAH metabolism. Functional hypotheses were raised for several of the *C. teleta* CYPs. CYP51A1 is very likely to catalyze the production of cholesterol, due to a ∼65% amino acid identity and clear orthology to other CYP51 sequences. Yet, the steroidogenic pathway was not completely identified. Cholesterol side-chain-cleavage has been hypothesized as the function of CYP376A1. Still, there are no obvious candidates for 17α-hydroxylase and aromatase enzymes, which are carried out by CYP17A and CYP19A, respectively, in vertebrates. Considering that *C. teleta* produces *de novo* estradiol, these reactions are likely undertaken by other CYPs. Future studies on invertebrate steroidogenesis should focus on the CYPs with low copy number and phylogenetic positions close to vertebrate steroidogenic CYPs shown in this study. *In silico* protein folding and docking studies may provide important clues to narrow the number of candidates genes for steroidogenic CYPs and direct future functional studies.

## Methods

The *C. teleta* genome used for this study was version 1 of the assembly (Joint Genome Institute, University of California); the genome assembly had approximately 7.9x coverage with 21,042 scaffolds with a total size of 333.7 Mb. The EST database (National Center for Biotechnology Information, July 2012) had approximately 130,000 reads. The other sequences used in phylogenetic analyses were retrieved from the Cytochrome P450 web-page [12]. Many vertebrate sequences were used in the analyses, although there was a focus on *Danio rerio, Mus musculus*, and *Homo sapiens*; species which have had rigorous annotation of their CYPome [7], [12], [33]. A select number of CYP sequences from invertebrates were included: *Haliotis diversicolor* [12], *Crassostrea gigas* (NCBI), *Daphnia pulex* [10] and *Helobdella robusta* (JGI). For some phylogenies, *Caenorhabditis spp.* sequences were added [12].

### Gene annotation

The *Capitella teleta* EST database was assembled with PASA (r2012-06-25) [55], to align and extend the ESTs to each other and to align them to the *C. teleta* genome. Homology searching of ESTs was performed using all CYPs from human and zebrafish using tBLASTn (v2.2.27) [56]. Hits were compiled and the regions hit were autonomously counted via a custom Perl script. This approach allowed for many CYPs to be used as inputs for homology searches. Since CYPs can have <15% sequence identity from each other in their amino acid sequences, the use of a wide variety of CYPs during homology searching maximizes the number of unique hits. The hit regions were checked against the PASA outputs, overlaps collected, and approximate gene regions predicted. The putative gene regions were compared to previously annotated CYPs and gene boundaries were adjusted using Artemis (v14.0.0) [57] according to homology. Since there are no closely related species with their CYPome analyzed or a large EST/refseq database, exact exon boundaries were difficult to annotate with very high certainty. The exon boundaries were examined for appropriate splice signals [29] and to ensure that the boundaries were located appropriately to the reading frame. When there were large gaps in a gene, FASTA (36.3.5e) [58] searches against genome scaffolds were completed to find these missing regions, rather than BLAST, because FASTA has increased sensitivity.

Once annotated, CYPs were compared to the automated gene calls in September 2012 on JGI. There were no CYPs on JGI that were not found by the above method. The manual annotation made for more appropriate splice sites, with the JGI annotations at times leaving out segments or entire exons.

### Phylogenetic Analyses of CYP sequences

Alignments were created in MUSCLE (v3.8.31) [59] and manually refined in Mesquite (v2.75) [60] at the amino acid level. The N- and C-termini of CYPs are more divergent and were hard masked from further analysis. ZORRO (r2011-12-01) [61], a soft masking tool, was used on the remainder of the alignment. The phylogenetic analysis was conducted using a total of 220 sequences on RAxML (v7.4.2) [62] with 100 bootstraps using the slower algorithm (-b) with a gamma distribution. The clan 2 phylogeny had additional *C. elegans* sequences, the clan 3 and 4 phylogeny included from *Daphnia pulex, Daphnia magna* and *C. elegans* sequences, and the mitochondrial phylogeny incorporated sequences from *D. pulex, Caenorhabditis spp., and H. robusta*. The maximum likelihood analyses were based on the VT substitution model with fixed base frequencies (phylogeny of all clans), MTMAM substitution model with fixed base frequencies (clan 2 phylogeny), LG substitution model with empirical base frequencies (clan 3 and 4 phylogeny), or JTT substitution model with empirical base frequencies (mitochondrial clan phylogeny). The appropriate models were determined by ProtTest (v3.2) [63]. To root the phylogenetic trees, CYPs outside the clans were chosen; the clan 2 phylogeny used CYP family 504 genes from fungus (*Magnaporthe grisea* and *Nassarius fischeri*), the clan 3 and 4 phylogeny used CYP72s from *Arabidopsis thaliana*, and the mitochondrial clan phylogeny used CYP86s from *A. thaliana* and vertebrate CYP19s. These roots were selected based on closely related out-groups from David Nelson's "singlefam tree" on the Cytochrome P450 webpage [12].

All predicted *C. teleta* CYP genes were named by the cytochrome P450 nomenclature committee using the sequences provided, synteny data available and the phylogenetic trees generated in this study. STRAP (r2013-02-26) [64] was used for the motif work and Figtree (v1.4.0) was used to generate the figures of the phylogenetic trees.

Searches for the xenobiotic response element (XRE, TNGCGTG) [65] in the 10 kb upstream region of the predicted start site in each gene of families *CYP331, CYP4* and *CYP3061* used the MEME suite (v4.9.1) [66].

## Supporting Information

Table S1 **Cytochrome P450 superfamily complement in** ***Capitella teleta*** **.** Temporary names were based off the scaffold they were found on. Length is in amino acids. CYPs were named by the CYP nomenclature committee. There are a total of 84 full length CYPs. Only complete CYPs are listed.
(XLSX)

Table S2 **Incomplete cytochrome P450s in** ***Capitella teleta*** **.** Temporary names are based off the scaffold they were found on. The listed CYPs are not full length and are missing exons but have EST support.
(DOCX)

Table S3 **Cytochrome P450 fragments in** ***Capitella teleta*** **.** Temporary names are based off the scaffold they were found on. None of these fragments have EST support, except for p_342, suggesting they may be pseudogenes. P_342 had an early stop codon and is a pseudogene.
(DOCX)

Table S4 **Highly conserved motifs across the** ***Capitella teleta*** **CYPome.** Two motifs (K-helix, and meander coil) are represented in an aligned format to show conservation across the *C. teleta* CYPs. Bolded letters represent conserved residues. AA is the amino acid number where the motif begins in each gene. The expected motif sequence is given in each heading for comparison. The glutamic acid and arginine residues in the meander coil are conserved across the entire CYPome.
(DOCX)

Table S5 **Less conserved motifs across the** ***Capitella teleta*** **CYPome.** Two motifs (I-helix, and heme loop) are represented in an aligned format to show conservation across the *C. teleta* CYPs. Bolded letters represent conserved residues. AA is the amino acid number where the motif begins in each gene. The expected motif sequence is given in each heading for comparison. The cysteine residue in the heme loop are conserved across the entire CYPome. Note the lack of conservation in CYP372B1 (I-helix) and CYP3067A1 (I-helix and heme loop).
(DOCX)
