# Supplementary material for: The Cytochrome P450 Superfamily Complement (CYPome) in the Annelid Capitella teleta
Source: PLoS One. 2014 Nov 12;9(11):e107728. doi: 10.1371/journal.pone.0107728 (PMC4229089; doi:10.1371/journal.pone.0107728)
Supplement: Table S2 — Incomplete cytochrome P450s in Capitella teleta . Temporary names are based off the scaffold they were found on. The listed CYPs are not full length and are missing exons but have EST support. (DOCX) [file pone.0107728.s002.docx]

| Temporary Name | Region on Scaffold | Number of exons |
| --- | --- | --- |
| I_51a | 113563-114816 | 1 |
| I_91a | 178629-180233 | 7 |
| I_145c | 321636-322748 | 1 |
| I_226 | 133627-136799 | 10 |
| I_271b | 279012-281304 | 5 |
| I_396 | 103216-106894 | 5 |
| I_446 | 96010-97844 | 10 |
| I_458b | 77247-78132 | 2 |
| I_520 | 835384-85469 | 3 |
| I_881 | 61663-63036 | 2 |
| I_897 | 34082-35605 | 1 |
| I_3603 | 6001-7564 | 3 |
