# Supplementary material for: The Cytochrome P450 Superfamily Complement (CYPome) in the Annelid Capitella teleta
Source: PLoS One. 2014 Nov 12;9(11):e107728. doi: 10.1371/journal.pone.0107728 (PMC4229089; doi:10.1371/journal.pone.0107728)
Supplement: Table S3 — Cytochrome P450 fragments in Capitella teleta . Temporary names are based off the scaffold they were found on. None of these fragments have EST support, except for p_342, suggesting they may be pseudogenes. P_342 had an early stop codon and is a pseudogene. (DOCX) [file pone.0107728.s003.docx]

| Temporary Name | Region on Scaffold |
| --- | --- |
| P_5 | 212521-213095 |
| P_342 | 50262-54509 |
| P_371 | 38074-39314 |
| P_720 | 57387-56719 |
| P_1095 | 8791-7856 |
| P_2211 | 8299-8873 |
| P_5575 | 3510-4550 |
| P_7309 | 871-13 |
| P_8508 | 5096-4716, 3186-2401 |
| P_10760 | 1-306 |
| P_10990 | 334-1402 |
| P_16088 | 846-2089 |
| P_36404 | 2-505 |
