# Supplementary material for: The Cytochrome P450 Superfamily Complement (CYPome) in the Annelid Capitella teleta
Source: PLoS One. 2014 Nov 12;9(11):e107728. doi: 10.1371/journal.pone.0107728 (PMC4229089; doi:10.1371/journal.pone.0107728)
Supplement: Table S4 — Highly conserved motifs across the Capitella teleta CYPome. Two motifs (K-helix, and meander coil) are represented in an aligned format to show conservation across the C. teleta CYPs. Bolded letters represent conserved residues. AA is the amino acid number where the motif begins in each gene. The expected motif sequence is given in each heading for comparison. The glutamic acid and arginine residues in the meander coil are conserved across the entire CYPome. (DOCX) [file pone.0107728.s004.docx]

| CYP | K-helix | Meander Coil | |  |
| --- | --- | --- | --- | --- |
| AA | **EXXR** | **AA** | **FDPER** |  |
| CYP10B1 | 341 | ETFR | 393 | FKPER |
| CYP20A1 | 338 | ESLR | 390 | FDPER |
| CYP26D1 | 348 | EVLR | 400 | FDPDR |
| CYP26E1 | 378 | EVLR | 430 | FNPDR |
| CYP3052A1 | 362 | ELLR | 415 | FEPER |
| CYP3052A10 | 365 | ELLR | 418 | FQPER |
| CYP3052A11 | 352 | ELLR | 405 | FQPER |
| CYP3052A12 | 362 | ELLR | 415 | FQPER |
| CYP3052A13 | 362 | ELLR | 415 | FHPER |
| CYP3052A2 | 361 | ELLR | 414 | FEPER |
| CYP3052A3 | 361 | ELLR | 414 | FEPER |
| CYP3052A4 | 349 | ELLR | 378 | ..PER |
| CYP3052A5 | 363 | ELLR | 416 | FLPER |
| CYP3052A6 | 363 | ELLR | 416 | FQPER |
| CYP3052A7 | 360 | ELLR | 413 | FQPER |
| CYP3052A8 | 364 | ELLR | 417 | FQPER |
| CYP3052A9 | 364 | ELLR | 417 | FQPER |
| CYP3052B1 | 360 | ELLR | 413 | FQPER |
| CYP3052B1 | 365 | ELLR | 418 | FRPER |
| CYP3052B2 | 365 | ELLR | 418 | FRPER |
| CYP3052B3 | 364 | ELLR | 417 | FRPER |
| CYP3052B4 | 365 | ELLR | 418 | FRPER |
| CYP3052B5 | 357 | EILR | 410 | FKPER |
| CYP3052B6 | 357 | EILR | 410 | FKPER |
| CYP3052C1 | 356 | ELLR | 409 | FRPDR |
| CYP3052C1 | 360 | ELLR | 413 | FKPER |
| CYP3052D1 | 357 | ELLR | 410 | FRPER |
| CYP3052D2 | 357 | ELLR | 410 | FRPER |
| CYP3053A1 | 356 | ELTR | 409 | FKPER |
| CYP3054A1 | 360 | EIMR | 413 | FRPER |
| CYP3054A2 | 359 | EIMR | 412 | FRPER |
| CYP3054A3 | 359 | EIMR | 412 | FRPER |
| CYP3054A4 | 359 | EIMR | 412 | FRPER |
| CYP3054A5 | 359 | EIMR | 412 | FRPER |
| CYP3055A1 | 352 | ELLR | 405 | FIPER |
| CYP3055B1 | 354 | ELFR | 407 | FNPER |
| CYP3056A1 | 358 | ETLR | 411 | FNPNR |
| CYP3057A1 | 342 | ENQR | 395 | FRPER |
| CYP3058A1 | 368 | EVQR | 421 | FNPER |
| CYP3058A2 | 357 | EVQR | 410 | FNPER |
| CYP3058A3 | 351 | EVQR | 404 | FNPER |
| CYP3058B1 | 366 | EIQR | 419 | FRPGR |
| CYP3058C1 | 369 | EIQR | 422 | FRPER |
| CYP3059A1 | 356 | EIQR | 409 | FDPSR |
| CYP3059A2 | 356 | EIQR | 409 | FDPSR |
| CYP3059A3 | 349 | EIQR | 402 | FNPSR |
| CYP3060A1 | 356 | ELLR | 409 | FKPER |
| CYP3061A1 | 351 | EVAR | 404 | FRPER |
| CYP3062A1 | 359 | EVYR | 412 | FNPDN |
| CYP3062A2 | 372 | EVYR | 425 | FNPNR |
| CYP3063A1 | 358 | EIMR | 411 | FNPDR |
| CYP3064A1 | 335 | EVLR | 388 | FNPSR |
| CYP3065A1 | 379 | ETYR | 432 | FRPER |
| CYP3065A2 | 336 | ETYR | 389 | FRPER |
| CYP3065A3 | 336 | ETYR | 389 | FRPER |
| CYP3065A4 | 377 | ECYR | 430 | FKPER |
| CYP3065B1 | 375 | ETFR | 428 | FKPER |
| CYP3066A1 | 374 | ESLR | 426 | FDPER |
| CYP3066A2 | 374 | ESLR | 426 | FDPER |
| CYP3066A3 | 378 | ESLR | 430 | FIPER |
| CYP3066B1 | 358 | ESLR | 410 | FDPDR |
| CYP3066C1 | 380 | ESLR | 432 | FNPKR |
| CYP3067A1 | 344 | ESFR | 400 | FKYDR |
| CYP3068A1 | 336 | EMLR | 388 | FDPYR |
| CYP3069A1 | 312 | ETLR | 364 | FNPDQ |
| CYP3070A1 | 366 | ETLR | 418 | FNPDR |
| CYP3071A1 | 372 | EVQR | 424 | FDPGR |
| CYP3072A1 | 363 | ETLR | 416 | FNPDR |
| CYP331A1 | 405 | ETLR | 460 | FEPER |
| CYP331A2 | 363 | ETLR | 418 | FEPER |
| CYP331A3 | 401 | ETLR | 456 | FEPER |
| CYP331B1 | 407 | ETLR | 462 | FEPER |
| CYP362B1 | 345 | ESQR | 397 | FIPER |
| CYP371B1 | 403 | EALR | 455 | FIPER |
| CYP372A1 | 346 | ESFR | 391 | FIPER |
| CYP372B1 | 341 | ESFR | 394 | YHPER |
| CYP376A1 | 334 | EVLR | 386 | FKPER |
| CYP39B1 | 347 | ESIR | 398 | FKPDR |
| CYP44C1 | 359 | EGFR | 411 | FIPER |
| CYP4AT1 | 356 | ESLR | 409 | YDPER |
| CYP4BK4 | 304 | ESMR | 356 | FRPDR |
| CYP4EE1 | 399 | ESLR | 452 | YNPER |
| CYP4V25 | 367 | ETLR | 419 | FIPDR |
| CYP51A1 | 364 | ETLR | 416 | FNPDR |
