# Supplementary material for: The Cytochrome P450 Superfamily Complement (CYPome) in the Annelid Capitella teleta
Source: PLoS One. 2014 Nov 12;9(11):e107728. doi: 10.1371/journal.pone.0107728 (PMC4229089; doi:10.1371/journal.pone.0107728)
Supplement: Table S5 — Less conserved motifs across the Capitella teleta CYPome. Two motifs (I-helix, and heme loop) are represented in an aligned format to show conservation across the C. teleta CYPs. Bolded letters represent conserved residues. AA is the amino acid number where the motif begins in each gene. The expected motif sequence is given in each heading for comparison. The cysteine residue in the heme loop are conserved across the entire CYPome. Note the lack of conservation in CYP372B1 (I-helix) and CYP3067A1 (I-helix and heme loop). (DOCX) [file pone.0107728.s005.docx]

| CYP | I-helix  AA | [A/G]GX[D/E]T[T/S] | Heme Loop | |
| --- | --- | --- | --- | --- |
|  |  |  | **AA** | **PFXXGXRXCXG** |
| CYP10B1 | 284 | GAVETT | 415 | PFGHGARMCIG |
| CYP20A1 | 281 | AGFHTT | 410 | PFGFGKRKCLG |
| CYP26D1 | 289 | AGYETT | 421 | PFGSGSRSCAG |
| CYP26E1 | 317 | AGHLPT | 459 | PFGSGQRSCVA |
| CYP3052A1 | 307 | AGTATT | 440 | PFGAGPRVCLG |
| CYP3052A10 | 304 | AGTATT | 443 | PFGAGPRVCLG |
| CYP3052A11 | 305 | AGTATT | 430 | PFGAGPRVCLG |
| CYP3052A12 | 306 | AGTATT | 440 | PFGAGLRVCLG |
| CYP3052A13 | 306 | AGTATT | 440 | PFGAGPRVCLG |
| CYP3052A2 | 307 | AGTATT | 439 | PFGAGPRVCMG |
| CYP3052A3 | 308 | GGTATT | 439 | PFGAGPRVCLG |
| CYP3052A4 | 295 | AGTSTT | 401 | PFGAGPRVCLG |
| CYP3052A5 | 305 | AGTSTT | 441 | PFGAGPRVCLG |
| CYP3052A6 | 303 | AGTATT | 441 | PFSAGPRVCLG |
| CYP3052A7 | 304 | AGTATT | 438 | PFSAGPRVCLG |
| CYP3052A8 | 305 | AGTATT | 442 | PFGAGPRVCLG |
| CYP3052A9 | 305 | AGTSTT | 442 | PFGAGPRVCLG |
| CYP3052B1 | 303 | AGTTTT | 438 | PFGAGPRLCIG |
| CYP3052B1 | 308 | GATTTT | 443 | PFGAGTRVCLG |
| CYP3052B2 | 308 | AGTTTT | 443 | PFGAGTRVCVG |
| CYP3052B3 | 307 | AGTGTT | 442 | PFGAGTRVCIG |
| CYP3052B4 | 308 | AGTGTT | 443 | PFGAGTRVCIG |
| CYP3052B5 | 300 | AGTSTT | 435 | PFGAGPRVCVG |
| CYP3052B6 | 300 | AGTSTT | 435 | PFGAGPRVCVG |
| CYP3052C1 | 299 | AGTGTT | 434 | AFGAGARVCIG |
| CYP3052C1 | 303 | AGTSTT | 438 | TFGGGQRKCIG |
| CYP3052D1 | 300 | AGTSTT | 435 | PFGAGTRVCLG |
| CYP3052D2 | 300 | AGTSTT | 435 | PFGAGTRVCLG |
| CYP3053A1 | 299 | AGVGTF | 434 | AYGAGQRVCLG |
| CYP3054A1 | 304 | AGVLTT | 438 | PFGMGPRICAG |
| CYP3054A2 | 303 | AGVLST | 437 | PFGMGPRICAG |
| CYP3054A3 | 303 | AGVLTT | 437 | PFGMGPRICAG |
| CYP3054A4 | 303 | AGVLTT | 437 | PFGMGPRICAG |
| CYP3054A5 | 303 | AGVLTT | 437 | PFGLGPRICAG |
| CYP3055A1 | 296 | AGIVTT | 430 | AFGAGPRMCVG |
| CYP3055B1 | 297 | AGSVST | 432 | PFSAGPRVCMG |
| CYP3056A1 | 301 | SGTLTS | 436 | PFGAGRRMCLG |
| CYP3057A1 | 286 | AGIDTI | 416 | PFAGGRRVCLG |
| CYP3058A1 | 312 | AGAETT | 441 | PFSVGPRMCAG |
| CYP3058A2 | 301 | AGAETT | 430 | PFSVGPRMCAG |
| CYP3058A3 | 295 | AGGETT | 424 | PFGVGPRMCAG |
| CYP3058B1 | 310 | AGADTS | 439 | NFGIGKWSCPG |
| CYP3058C1 | 313 | AGSVTT | 442 | PYGIGPRACAG |
| CYP3059A1 | 300 | AGTESS | 429 | PFGIGRRVCLG |
| CYP3059A2 | 300 | AGTETT | 429 | PFGIGRRVCLG |
| CYP3059A3 | 293 | AGTETT | 422 | PFGIGRRLCLG |
| CYP3060A1 | 299 | AGLDIV | 432 | PYGMGRRRCIG |
| CYP3061A1 | 289 | AGADTV | 426 | MFGYGKRRCIG |
| CYP3062A1 | 306 | AGVESM | 434 | PFGYGMRRCPG |
| CYP3062A2 | 319 | AGTESM | 447 | PFGAGMRRCPG |
| CYP3063A1 | 301 | AGTETS | 437 | PFGAGKRKCIG |
| CYP3064A1 | 281 | GVSDGS | 410 | PFSTGQRSCVG |
| CYP3065A1 | 322 | DSLDTL | 452 | PFGVGPRSCPG |
| CYP3065A2 | 279 | DSLDTL | 409 | PFGVGPRSCPG |
| CYP3065A3 | 279 | DSLDTL | 409 | PFGVGPRSCVG |
| CYP3065A4 | 320 | DALDSL | 450 | PFGLGPRACAG |
| CYP3065B1 | 318 | DSADTL | 448 | PFGLGPRACLG |
| CYP3066A1 | 317 | NAFSTI | 446 | PFGFGPRNCVG |
| CYP3066A2 | 317 | AAFGTI | 446 | PFGFGPRNCVG |
| CYP3066A3 | 321 | AAYGTI | 450 | PFGQGPRHCIA |
| CYP3066B1 | 302 | AGYSTI | 430 | PFGFGPRHCIG |
| CYP3066C1 | 323 | SGHSTV | 452 | PFGMGPRSCIG |
| CYP3067A1 | 286 | ....NT | 424 | AFGS...LCPG |
| CYP3068A1 | 278 | ASQETL | 411 | PFGAGNRTCVG |
| CYP3069A1 | 264 | GAQETL | 383 | PFGGGAHACVG |
| CYP3070A1 | 309 | AGQETT | 436 | PFSLGQRSCLG |
| CYP3071A1 | 314 | AGHETT | 442 | PFSTGPHKCLG |
| CYP3072A1 | 306 | AGHETT | 435 | PFLIGPRMCLG |
| CYP331A1 | 348 | AGYETT | 480 | PFGAGPRNCIG |
| CYP331A2 | 306 | AGYDTT | 438 | PFGAGPRNCIG |
| CYP331A3 | 344 | AGFETS | 476 | PFGVGPRNCMG |
| CYP331B1 | 350 | AGFETT | 482 | PFGAGPRNCVG |
| CYP362B1 | 288 | AGVDTT | 421 | PFGHGARSCIG |
| CYP371B1 | 346 | GAVDTT | 476 | PFGFGARSCIG |
| CYP372A1 | 281 | AGIDST | 414 | PFGYGPRMCIG |
| CYP372B1 | 280 | PNIEIEDRST | 421 | PFSHGLRACPG |
| CYP376A1 | 277 | AAVDTT | 409 | PFGFGTRMCLG |
| CYP39B1 | 282 | ASLANA | 419 | PFGGGRFQCPG |
| CYP44C1 | 301 | DGMITT | 432 | PFSCGPRMCPG |
| CYP4AT1 | 299 | EGHDTT | 429 | PFSAGPRNCIG |
| CYP4BK4 | 245 | EGHDTT | 376 | PFSAGPRNCIG |
| CYP4EE1 | 340 | EGHDTT | 472 | PFSAGPRNCIG |
| CYP4V25 | 308 | EGHDTT | 439 | PFSAGLRNCIG |
| CYP51A1 | 306 | AGQHTS | 437 | PFGAGRHRCIG |
